# Stage Two Malignant Melanoma of the Clitoris: A Case Report

**DOI:** 10.7759/cureus.4530

**Published:** 2019-04-23

**Authors:** Bradley M White, Alysha Vartevan, Jenni Harris, Crystal Scott, Daryl Eber

**Affiliations:** 1 Interventional and Diagnostic Radiology, Larkin Community Hospital, Miami, USA; 2 Radiology, Barrow Neurological Institute, Phoenix, USA; 3 Radiology, University of Wisconsin, Madison, USA; 4 Radiology, University of California Davis Medical Center, Davis, USA; 5 Nuclear Medicine, University of Miami, Miami, USA

**Keywords:** melanoma, lymphoscintigraphy, nuclear medicine

## Abstract

Mucosal melanomas are rare in comparison to cutaneous melanomas, accounting for approximately 1% of all melanoma cases. Vulvovaginal melanoma is the least common mucosal melanoma subtype. For patients without distant metastases at presentation, regional lymph node involvement is the most important prognostic indicator. Lymphoscintigraphy is a method used to identify the sentinel lymph node (SLN), directing subsequent biopsies to the lymph node at highest risk for cancer spread.

We present a 67-year-old Hispanic female with stage II B (T4aN0M0) melanoma of the clitoris. The patient initially sought medical treatment for a pigmented vulvar lesion over her clitoris that occasionally bleeds. She stated that over the past month the lesion began to grow peripherally and then acquired a very dark color. The patient underwent local excision of the mass and subsequent pathology revealed malignant melanoma. The patient underwent lymphoscintigraphy which allowed localization and subsequent biopsy of the left inguinal sentinel node. The left inguinal sentinel node was negative for metastatic melanoma. It was opted to keep her under observation.

Compared to cutaneous melanoma, patients with mucosal melanoma usually present with more advanced disease, and thus efficacious imaging practices play a significant role in the management of the disease. Lymphoscintigraphy is a well-tolerated, validated, cost-effective, and reliable method of detecting sentinel nodes with minimal radiation exposure to the patient. In our case, early clinical detection allowed for prompt surgical intervention, pathologic diagnosis, and reliable staging via lymphoscintigraphy of a rare form of malignant melanoma.

## Introduction

Mucosal melanomas are rare in comparison to cutaneous melanomas, accounting for approximately 1% of all melanoma cases [1]. Mucosal melanomas may arise from the epithelium lining the respiratory, alimentary, or urogenital tract. Vulvovaginal melanoma is the least common mucosal melanoma subtype, composing 18% of mucosal melanoma cases and less than 0.2% of all melanoma cases [1-3].

For patients without distant metastases at presentation, regional lymph node involvement is the most important prognostic indicator [4]. Lymphoscintigraphy is a method used to identify the sentinel lymph node (SLN), directing subsequent biopsies to the lymph node at highest risk for cancer spread [5]. Positive regional lymph node involvement indicates at least stage three disease, negatively affecting prognosis, and may make the patient a candidate for adjuvant immunotherapy and/or clinical trials with possible improvement in survival [6]. 

## Case presentation

We present a 67-year-old Hispanic female with stage II B (T4aN0M0) melanoma of the clitoris. The patient initially sought medical treatment for her condition in January 2015 at which time her chief complaint was a pigmented vulvar lesion over her clitoris that occasionally bleeds. She stated that over the past month the lesion began to grow peripherally and then acquired a very dark color. The patient's past medical history included hypothyroidism, hypertension, hyperlipidemia, and gastritis. Her past surgical history included total hysterectomy and left breast lumpectomy of a benign cyst. 

**Figure 1:**
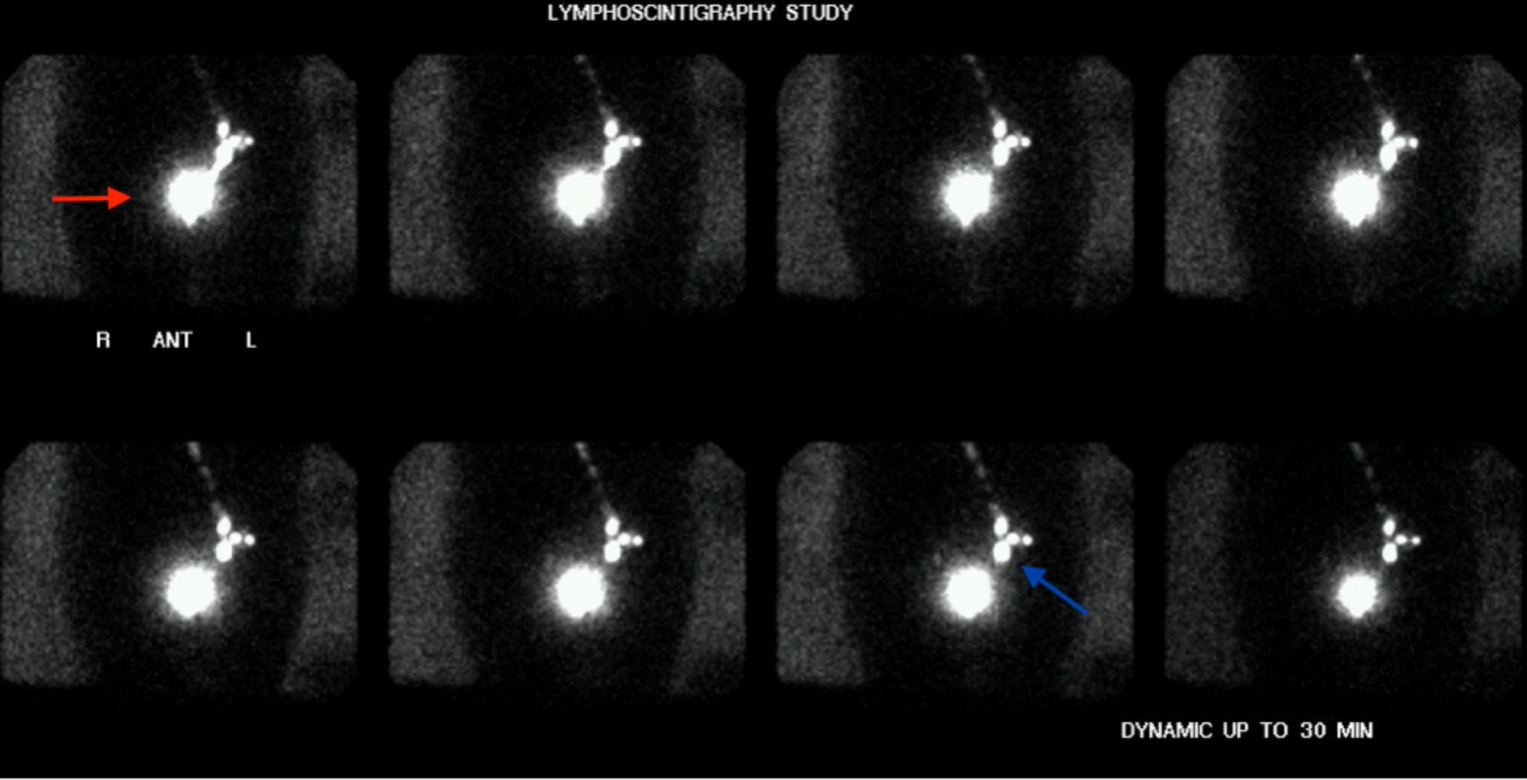
Lymphoscintigraphy study, anterior images Injection of technetium 99 m sulfur colloid at the site of melanoma lesion (red arrow). Dynamic anterior images demonstrate the filling of three lymph nodes in the left groin via a single lymphatic channel. The inferior node (blue arrow) is the SLN (sentinel lymph node); the superior nodes are secondary nodes.

**Figure 2:**
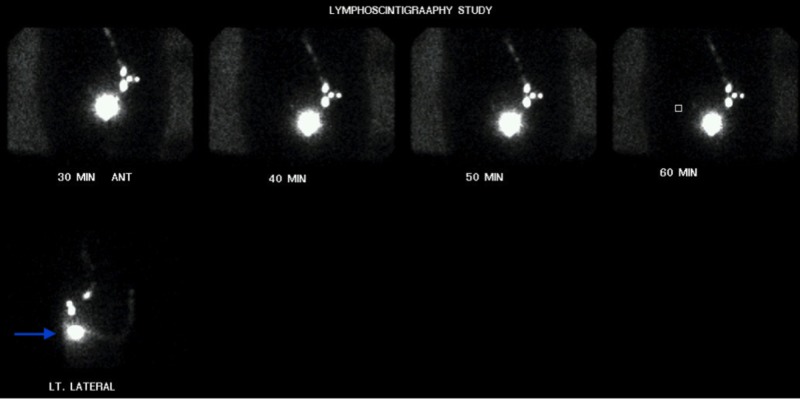
Lymphoscintigraphy study, delayed anterior and lateral views Delayed anterior and lateral emission and transmission images of the pelvic region revealing the anterior location of the sentinel node (arrow).

The patient underwent local excision of the mass and subsequent pathology revealed malignant melanoma thickness: 8.0 mm; invading reticular dermis; ulceration: none; margins: free of involvement; lymphovascular or perineural invasion: none; and microsatellites: none. The patient underwent a re-excision on February 5, 2015, which was negative for melanoma. A staging positron emission tomography (PET) was negative for metastasis on March 11, 2015. On April 2, 2015, the patient underwent lymphoscintigraphy, which allowed localization and subsequent biopsy of the left inguinal SLN (Figures 1-2). The left inguinal SLN was negative for metastatic melanoma, confirmed with immunohistochemical stains for Malan-A and S-100. Treatment options presented by the patient’s oncologist included observation or clinical trial of high dose interferon for one year. Because the patient had good pathological tumor features, was lymph node negative, and had social environment limitations, and considering that interferon therapy has no proven survival benefit, it was opted to keep her under observation.

On a six-month routine follow-up, the patient reported that for the past month she felt 'as if she has a piece of paper hanging from her vagina', and a constant burning pain on the anterior surface of her vulva and dyspareunia; however, physical exam showed no sign of recurrence and she was referred to gynecology with negative pap smear and mammogram. At one-year follow-up on April 26, 2016, the patient remained asymptomatic; repeat PET scan was negative, and no local inguinal nodes or masses were noted.

## Discussion

Compared to cutaneous melanoma, patients with mucosal melanoma usually present with more advanced disease and thus efficacious imaging practices play a significant role in the management of the disease.

All vulvar melanomas are currently staged using the same TNM staging system for cutaneous melanomas proposed by the American Joint Committee on Cancer (AJCC). Primary tumor size is represented by (T), which ranges from 0 (no evidence of primary tumor) to 4 (>4 mm), with 'a' indicating no ulceration. Regional lymph nodes are denoted as (N), ranging from 0 (no regional metastases detected) to 3 (four or more metastatic lymph nodes). Distant metastasis is symbolized by (M), which is either 0 (absent) or 1 (present). Based on an evaluation of the TNM staging system, patients are divided into four stages, which provide prognostic information. Stage 1 includes T1a through T2a, stage 2 includes tumors with a higher risk of recurrence (T2b through T4b) but do not have any evidence of metastases. Stage 3 designates any involved lymph nodes and stage 4 represents distant metastases. The five-year relative survival rate as a function of stage of disease at presentation is as follows: stage 1 (70%), stage 2 (50%), stage 3 (48%) and stage 4 (24%) [7].

Lymphoscintigraphy is a well-tolerated, validated, cost-effective, and reliable method of detecting sentinel nodes with minimal radiation exposure to the patient [8]. A radiolabeled tracer, such as technetium 99 m sulphur colloid, is injected intradermally within 1.5 cm of the primary lesion. The tracer is subsequently picked up by local lymphatic channels and is then deposited within the nodal tissue receiving the lymphatic flow from the injection site. The SLN is frequently detected within 10 to 30 minutes (Figure 1).

## Conclusions

In our case, early clinical detection allowed for prompt surgical intervention, pathologic diagnosis, and reliable staging via lymphoscintigraphy of a rare form of malignant melanoma.
